# Extracellular Matrix–Associated Biomarkers for Hepatocellular Carcinoma: Insights From Machine Learning and Single‐Cell Analysis

**DOI:** 10.1155/ijog/6654142

**Published:** 2026-02-24

**Authors:** Pedram Asadi Sarabi, Elham Rismani, Amir Ali Judaki, Amirhossein Farrokhzad, Zahra Hendi, Moustapha Hassan, Massoud Vosough

**Affiliations:** ^1^ Department of Regenerative Medicine, Cell Science Research Center, Royan Institute for Stem Cell Biology and Technology, ACECR, Tehran, Iran, acecr.ac.ir; ^2^ Molecular Medicine Department, Biotechnology Research Center (BRC), Pasteur Institute of Iran, Tehran, Iran, pasteur.ac.ir; ^3^ Department of Cellular and Molecular Biology, Faculty of Sciences and Advanced Technology in Biology, University of Science and Culture, Tehran, Iran, usc.ac.ir; ^4^ Experimental Cancer Medicine, Institution for Laboratory Medicine, and Karolinska University Hospital, Karolinska Institute, Stockholm, Sweden, ki.se

**Keywords:** extracellular matrix remodeling, hepatocellular carcinoma, machine learning, overall survival, systems-level biomarker

## Abstract

The 5‐year overall survival rate for hepatocellular carcinoma (HCC) patients remains below 20%. Alterations in the extracellular matrix (ECM) are increasingly recognized as central drivers of HCC initiation and progression. This study applied a system biology framework integrating omics data and machine learning to analyze gene expression and regulatory networks in HCC using The Cancer Genome Atlas. Eight ECM‐associated genes (*CSPG4*, *CD34*, *C1orf35*, *ESM1*, *MAPT*, *PLXDC1*, *STC2*, and *THBS4*) were identified as upregulated diagnostic biomarkers with strong discriminatory power. Among them, *MAPT*, *PLXDC1*, and *STC2* showed significant associations with poor overall survival, defining a prognostic subset. Validation in the GSE104310 and GSE144269 datasets confirmed consistent expression patterns across cohorts. Functional enrichment linked these genes to tissue remodeling and angiogenesis. Single‐cell RNA sequencing revealed *MAPT* upregulation in T cells, *PLXDC1* enrichment in cancer‐associated fibroblasts, and mild *STC2* elevation in tumor‐associated macrophages and endothelial cells. These findings identify key ECM‐based biomarkers with potential for early detection, prognosis, and therapeutic targeting in HCC.

**Summary**

**Hepatocellular carcinoma (HCC)**
• The most common type of liver cancer, representing 75%–85% of all cases, is characterized by a poor prognosis and low 5‐year survival rates.
**Liver hepatocellular carcinoma (LIHC)**
• Alternative term used interchangeably with HCC.
**Five-year survival rate**
• The proportion of patients who remain alive 5 years after diagnosis for HCC, this rate is approximately 18%.
**Liquid biopsy**
• A noninvasive diagnostic method that analyzes biomolecules present in blood or other bodily fluids to identify and monitor tumors
**Extracellular matrix (ECM)**
• A complex network of proteins and carbohydrates supportive for surrounding cells that play a significant role in tumor progression and metastasis.
**Differentially expressed genes (DEGs)**
• Genes that show statistically significant different expression levels between different conditions, such as comparing tumor tissues to normal tissues.
**MicroRNA (miRNA)**
• Small noncoding RNA molecules that regulate gene expression of target mRNAs, influencing a range of biological processes.
**Machine learning classifier**
• An algorithm that uses statistical techniques to classify data into different categories.
**Gene Ontology analysis**
• A method used to categorize genes based on their functions, providing insights into the biological processes they are involved in.
**Receiver operating characteristic (ROC) curve**
• A graphical representation used to assess the performance of a binary classifier system, illustrating the trade‐off between sensitivity and specificity.
**Area under the curve (AUC)**
• A measure of the accuracy of a classifier; higher AUC values indicate better performance in distinguishing between different classes.

## 1. Introduction

Liver hepatocellular carcinoma (LIHC) or hepatocellular carcinoma (HCC) is the most common form of liver cancer, accounting for 75%–85% of cases of liver cancers. The 5‐year survival rate for patients with HCC is only 18% [1]. According to the newest Global Cancer Statistics, there have been 905,677 new cases of liver cancer and 830,180 new deaths reported worldwide [2]. Despite a decrease in incidence, liver cancer remains a primary worldwide health concern, with an estimated one million cases by 2025 [3]. However, hepatectomy, liver transplantation, radiotherapy, chemotherapy, and targeted therapy have improved the clinical prognosis for some HCC patients; the prognosis of HCC remains dismal, with a postoperative recurrence rate of up to 70% [4]. Undetectable progression in the early stage and the possibility of metastasis in the advanced stage led to late diagnosis and poor prognosis [5]. Therefore, the discovery of novel biomarkers and prognostic prediction models holds great clinical significance, as it improves diagnostic accuracy.

Liquid biopsy, a noninvasive method, has recently been developed for the detection and monitoring of tumors by analyzing biomolecules obtained from blood circulation and other bodily fluids [6]. This approach offers a deeper insight into tumor heterogeneity across various sites and can aid in tracking responses to treatment [7]. Liquid biopsies contain valuable information such as DNA mutations, copy number alterations (CNAs) [8], transcriptome/proteome profiling [9], epigenetic changes [10], and metabolite profiling [11]. Despite its advantages, liquid biopsy has limitations, such as low sensitivity in the early detection of HCC [12, 13]. Therefore, precise phenotyping and genotyping of liquid biopsy samples are essential [14].

The extracellular matrix (ECM) is a complex and dynamic network of secreted macromolecular compounds. ECM provides structural support to regulate essential cellular functions, such as growth, migration, differentiation, and apoptosis [15]. Accumulating evidence has suggested that alterations in the structure and composition of the ECM occur during tumor development and progression. Disruptions of the ECM enable cancerous cells to gain multiple hallmarks of malignancy [16]. Different tumors are associated with a distinct ECM signature directing aspects of tumor behavior, including tumor aggression, metastasis, and treatment sensitivity [17–19]. ECM dynamicity is dysregulated by several conditions, including aging, hypoxia, injury, inflammatory diseases, and cancer [20]. This impacts growth factor signaling pathway activity, inhibition of tumor suppressors, and promotion of angiogenesis, and these might represent the main effectors of tumor growth ([21]). Interestingly, a dynamic ECM has been associated with HCC carcinogenesis, progression, and prognosis [22].

In this study, ECM‐related genes are developed as novel biomarkers for predicting the prognosis of HCC using data from a total of 421 patients from The Cancer Genome Atlas (TCGA) database. We then validated their prognostic prediction capacity using data from the GEPIA database, demonstrating a correlation with patients’ survival rates. Additionally, Gene Ontology analysis suggested that these mRNAs were most significantly enriched in terms of tissue remodeling, regulation of peptidyl‐tyrosine phosphorylation, angiogenesis, and integrin binding. Moreover, single‐cell RNA sequencing (scRNA‐seq) analysis of the liver samples showed valuable insights into the gene expression patterns across normal, adjacent, and cancerous liver tissues. The identified biomarkers are anticipated to provide a noninvasive early detection model for HCC patients.

## 2. Methods

### 2.1. Data Retrieval

TCGA (http://cancergenome.nih.gov) is a publicly funded project that provides multidimensional data on multiple cancer types at the DNA, RNA, and protein levels. The project encompasses mRNA data from 421 patients, including 371 primary tumors and 50 solid tissue standard specimens for integrated analysis. Moreover, miRNA data from 425 patients, including 375 primary tumors and 50 solid tissue standard specimens were used for the integrated analysis.

### 2.2. Identification of Differentially Expressed miRNAs and mRNAs

We performed a comprehensive analysis of the TCGA‐LIHC dataset, which focuses on LIHC, to identify DEGs. Using the GDCRNATools package in R, we retrieved RNA‐seq and miRNA data along with associated clinical information. The data underwent a series of preprocessing steps, including the removal of duplicated samples and the exclusion of nonprimary tumor or nonsolid standard tissue samples. To mitigate technical variations and ensure the consistency of expression levels across samples, the datasets were normalized using the gdcVoomNormalization function. Differential gene expression analysis was then conducted using the gdcDEAnalysis function with the Limma method, resulting in the identification of various subsets of DEGs, including both all miRNAs and protein‐coding genes.

### 2.3. Identification of the Optimal Diagnostic Biomarkers Based on the Machine Learning Approach

Gene expression data from TCGA‐LIHC were preprocessed by separating features (gene expressions) from targets (sample types), applying an 80/20 train/test split, and normalizing with StandardScaler. Multiple supervised classifiers were benchmarked using fivefold cross‐validation: random forest (RF; randomForest package), support vector machine with RBF kernel (SVM‐RBF; e1071), LASSO logistic regression (glmnet for feature selection followed by glm), *k*‐nearest neighbors (k‐NNs, *k* = 5), decision tree (rpart), and naive Bayes (e1071). All models were evaluated using accuracy, precision, recall, and *F*1 score. A RF model was trained using normalized TCGA expression data, and gene importance scores were calculated. Based on the kernel density distribution of feature importance values, a cutoff of 0.002 was chosen empirically from the right tail of the distribution to retain only the most informative genes. This threshold was used for feature selection, independent of differential expression *p* values. Genes surpassing this cutoff were further screened for ECM association using the DAVID database. Additionally, we highlighted the specific genes of interest and visualized their importance scores, providing insights into the potential diagnostic biomarkers.

### 2.4. Targeted Selection of ECM‐Linked Genes Through Differential Expression Analysis

The focus was on enhancing the specificity of the candidate genes by emphasizing their association with the ECM. The structural components of the ECM are dynamic and play a crucial role in cellular behavior and signaling. Considering the importance of secreted proteins in intercellular communication, we specifically chose genes that are either part of the ECM structure or closely associated with it through their interactions.

These genes are often implicated in critical cellular processes owing to their secretion into the extracellular space. To identify ECM‐related genes, we utilized the DAVID database (https://david.ncifcrf.gov/), which facilitated the selection of genes linked explicitly to the ECM. Additionally, this refined set of genes was further filtered based on their upregulation and *p* value ≤ 0.05, as determined by DEG analysis. This comprehensive approach is aimed at identifying genes that not only have high importance scores but are also significantly related to the ECM and exhibiting distinct expression patterns in LIHC, thus broadening the pool of potential diagnostic biomarkers.

### 2.5. Network of Differentially Expressed miRNAs and mRNAs

In this pivotal phase of our study, we conducted an in‐depth analysis of miRNA expression profiles obtained from TCGA samples. Concurrently, we leveraged the miRDB database (https://mirdb.org/) to identify potential target miRNAs corresponding to our previously identified candidate genes. The subsequent step involved the intersection of miRNAs from both groups, which led to the identification of shared miRNAs with putative regulatory roles over the candidate genes. To provide a comprehensive visual representation of these intricate regulatory connections, a network was constructed using Cytoscape software [23]. This network serves as a powerful tool for elucidating the connections between differentially expressed miRNAs and mRNAs, offering insights into the potential miRNA‐mediated regulatory networks influencing the expression of candidate genes associated with LIHC. The integration of miRNA–target interactions from miRDB enhances the robustness of this network, providing a more nuanced understanding of the regulatory landscape in LIHC.

### 2.6. miRNA Enrichment Analysis Using miRNA Enrichment Analysis and Annotation (miEAA) Database

To investigate the potential roles of miRNAs, their enrichment analysis was performed using the miEAA database (https://ccb-compute2.cs.uni-saarland.de/mieaa/). This database integrates miRNA expression profiles with the functional annotations to identify the enriched miRNA signatures associated with specific biological processes or pathways.

### 2.7. Gene–Gene Interaction Network

GeneMANIA database (https://genemania.org/) was applied to identify the genes that were most relevant to the query gene set and to construct a composite gene–gene functional interaction network.

### 2.8. ROC Curve Analysis

This section presents ROC curve analysis for a set of selected genes, evaluating their potential as biomarkers for LIHC. The ROC curves illustrate the sensitivity and specificity of each gene as a classifier. In contrast, the associated AUC values provide a quantitative measure of their overall performance. Notably, specific genes exhibit high AUC values, indicating robust discriminatory power between case and control samples. The ensuing discussion delves into the implications of these findings, exploring potential biological significance and outlining directions for further validation and clinical applications.

### 2.9. Correlation Analysis Between *α*‐Fetoprotein (AFP) Level and ECM‐Associated Gene Expression

To explore the association between AFP levels and ECM‐associated gene expressions, correlation analyses were conducted using TCGA‐LIHC clinical and transcriptomic data. Genes selected for analysis included *CSPG4*, *CD34*, *C1orf35*, *ESM1*, *MAPT*, plexin domain‐containing 1 (*PLXDC1*), stanniocalcin 2 (*STC2*), and *THBS4*. For each gene, the log2‐transformed AFP concentration (g/L + 1) was plotted against mRNA expression (RNA‐Seq TPM values). Both Spearman’s rank correlation coefficient (*ρ*) and Pearson’s correlation coefficient (*r*) were calculated to evaluate monotonic and linear associations, respectively. Scatter plots were generated in the cBioPortal platform (https://www.cbioportal.org/) to visualize gene–AFP correlations, and *p* < 0.05 was considered statistically significant.

### 2.10. External Validation Using GEO Cohorts (GSE104310 and GSE144269) and GEPIA

To enhance the reliability and generalizability of the identified ECM‐associated biomarkers, additional validation was conducted using independent transcriptomic cohorts. Two external RNA‐seq datasets, GSE104310 and GSE144269, were incorporated to evaluate expression consistency across distinct populations and sequencing platforms. GSE104310 includes paired tumor and adjacent nontumor tissues from HCC patients collected at Sun Yat‐sen University Cancer Center and sequenced using the Illumina HiSeq 2500 platform. GSE144269 comprises 70 paired tumor and nontumor samples from Mongolian HCC patients profiled by high‐throughput RNA sequencing. Raw expression matrices for both datasets were downloaded from GEO, normalized using the variance‐stabilizing transformation (VST), and log2 fold‐changes were computed for each of the eight ECM‐associated genes. These analyses enabled cross‐cohort comparison with TCGA‐LIHC and provided robust external validation of biomarker expression trends.

In parallel, the GEPIA database (http://gepia.cancer-pku.cn/) was used to independently examine differential expression patterns and survival associations through standardized box plots and Kaplan–Meier analyses. This multilayer validation strategy integrates population‐level datasets (TCGA), independent GEO cohorts (GSE104310 and GSE144269), and web‐based clinical resources (GEPIA), collectively strengthening the robustness and clinical relevance of the identified biomarkers.

### 2.11. scRNA‐seq Analysis

The scRNA‐seq data were obtained from the GEO under Accession Number GSE189903, comprising transcriptomic profiles of liver tissues from patients with HCC and adjacent normal tissue. The dataset included samples from the tumor core, the tumor border, and the adjacent nontumor regions. Raw data, consisting of a count matrix, gene annotations, cell barcodes, and metadata, were downloaded and processed using the Seurat package (v4.3.0) in R (v4.2.2). A Seurat object was generated by integrating the count matrix with metadata, and the dataset was subsequently divided into individual samples based on sample identifiers. For this analysis, four samples from adjacent nontumor tissue and six from the tumor core were selected based on the quality of each sample.

Quality control was applied to each sample, excluding cells with mitochondrial content exceeding 20% and restricting feature counts to within two standard deviations of the median per sample as detailed in Table S1. Data normalization employed the “LogNormalize” method with a scale factor of 10,000, followed by identification of the Top 2000 variable features using the VST approach. To maintain condition‐specific differences, normal and cancer samples were integrated separately using reciprocal principal component analysis (rPCA) with anchors derived from variable genes. The integrated datasets underwent scaling and PCA, with the first 10 principal components chosen based on elbow plot evaluation. Clustering was performed at a resolution of 0.2, and uniform manifold approximation and projection (UMAP) and *t*‐distributed stochastic neighbor embedding (*t*‐SNE) were used for visualization based on published cell annotations [24]. Expression levels of selected genes were assessed across clusters and conditions using violin plots.

## 3. Results

### 3.1. Differential Gene and miRNA Expression in HCC

The TCGA project provides extensive information on various cancers, including data for LIHC. A log‐based fold‐change greater than one and a *p* value less than 0.05 were considered the inclusion criteria. This led to the identification of 2123 DEGs and 454 miRNAs in primary tumors compared to the normal tissues. These findings demonstrated significant molecular differences between the tumor and normal samples, offering valuable insights into the complexities of HCC.

### 3.2. The Optimal Diagnostic Biomarkers Based on a Machine Learning Approach

To identify the most effective diagnostic biomarkers, we applied multiple supervised machine learning algorithms to normalized TCGA gene expression data. RF, SVM, LASSO, *k*‐NN, and decision tree classifiers all yielded high test performance, with accuracy ranging from 97.65% to 98.67% and *F*1 scores exceeding 98.6% (Table 1). These results confirm the strong discriminatory signal between tumor and standard samples in the dataset. Although model performance was comparable across classifiers, RF was selected for final feature selection due to its interpretability and ability to rank gene importance. Using a feature importance threshold of 0.002, we identified 127 informative genes. We then visualized these genes through scatter plots and kernel density distribution curves, highlighting their potential diagnostic relevance (Figure 1a,b).

**Table 1:** Comparison of machine learning models on TCGA‐LIHC data.

Model	Accuracy	Precision	Recall	*F*1 score
Random forest	97.65%	98.67%	98.67%	98.67%
SVM (RBF)	97.65%	98.67%	98.67%	98.67%
LASSO logistic Reg.	97.65%	98.67%	98.67%	98.67%
*k*‐NN (*k* = 5)	97.65%	100.00%	97.33%	98.65%
Decision tree	97.65%	100.00%	97.33%	98.65%
Naive Bayes	85.88%	87.95%	97.33%	92.41%

Figure 1Identification of optimal diagnostic biomarkers for liver hepatocellular carcinoma (LIHC). (a) Scatter plot showing random forest gene importance scores. (b) Kernel density plot displaying the distribution of importance values. (c) Venn diagram illustrating overlapping genes between protein‐coding genes and random forest–selected candidates.

**Figure:**
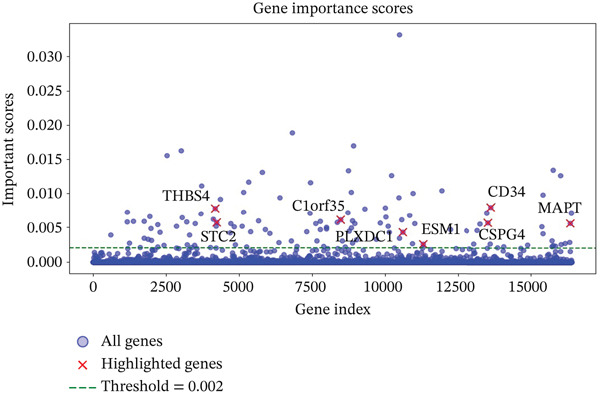
(a)

**Figure:**
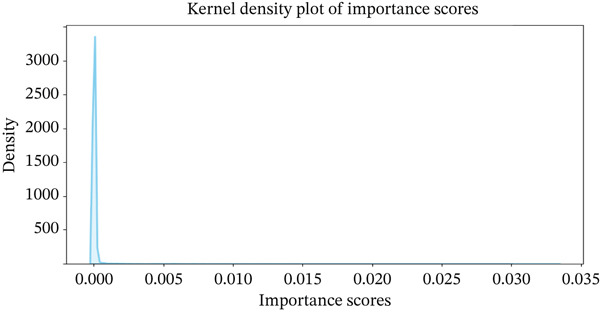
(b)

**Figure:**
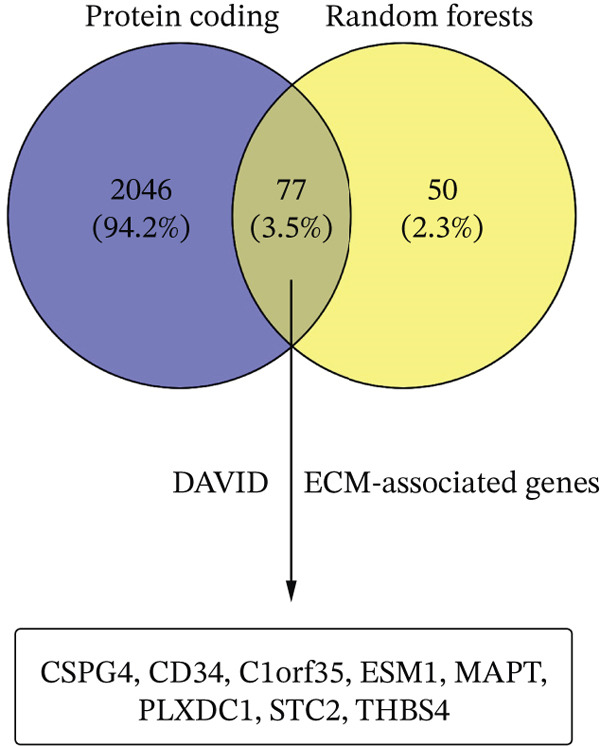
(c)

### 3.3. ECM‐Associated Biomarkers in LIHC

From the 2123 DEGs identified, a machine learning algorithm selected 127 genes, with 77 of these genes overlapping between the two groups under study (Figure 1c). Among these 77 genes, eight were linked to the ECM according to the DAVID database, and their expression levels were notably elevated. To refine the selection, we applied a strict feature importance cutoff of 0.002 derived from the RF model, retaining only genes with the highest discriminatory power. This filtering yielded eight ECM‐associated genes, *CSPG4*, *CD34*, *C1orf35*, *ESM1*, *MAPT*, *PLXDC1*, *STC2*, and *THBS4* (Table 2), which may serve as promising biomarkers for liver cancer.

**Table 2:** Log2FC values for TCGA, GSE104310, and GSE144269 datasets.

Gene	TCGA Log2FC	GSE104310 Log2FC	GSE144269 Log2FC
*CSPG4*	2.1914	1.7631	1.15
*CD34*	1.9888	2.0143	2.03
*C1orf35*	1.2209	0.9704	0.48
*ESM1*	4.1146	4.6985	3.82
*MAPT*	2.4873	1.8854	1.88
*PLXDC1*	2.5861	1.3278	3.15
*STC2*	2.7846	3.3338	3.15
*THBS4*	4.2029	3.0244	1.88

### 3.4. Network of Differentially Expressed miRNAs and mRNAs

In this phase, we explored miRNA profiles from TCGA, predicting target miRNAs for our candidate genes using the miRDB database. By identifying shared miRNAs between TCGA and our findings, we developed a visual network using Cytoscape. This network reveals potential regulatory connections and provides insights into how miRNAs influence gene expression in liver cancer (Figure 2a).

Figure 2Analysis of differentially expressed miRNAs and mRNAs in liver cancer. (a) Network visualization showing regulatory connections between differentially expressed miRNAs and mRNAs in HCC. (b) Word cloud of enriched functional categories associated with 97 differentially expressed miRNAs regulating the eight ECM‐associated candidate genes. Font size reflects enrichment magnitude, highlighting key pathways in HCC biology.

**Figure:**
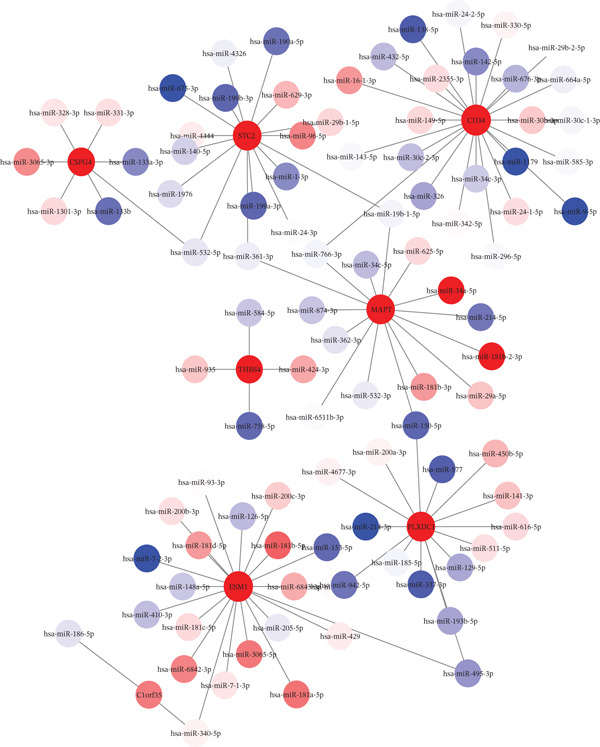
(a)

**Figure:**
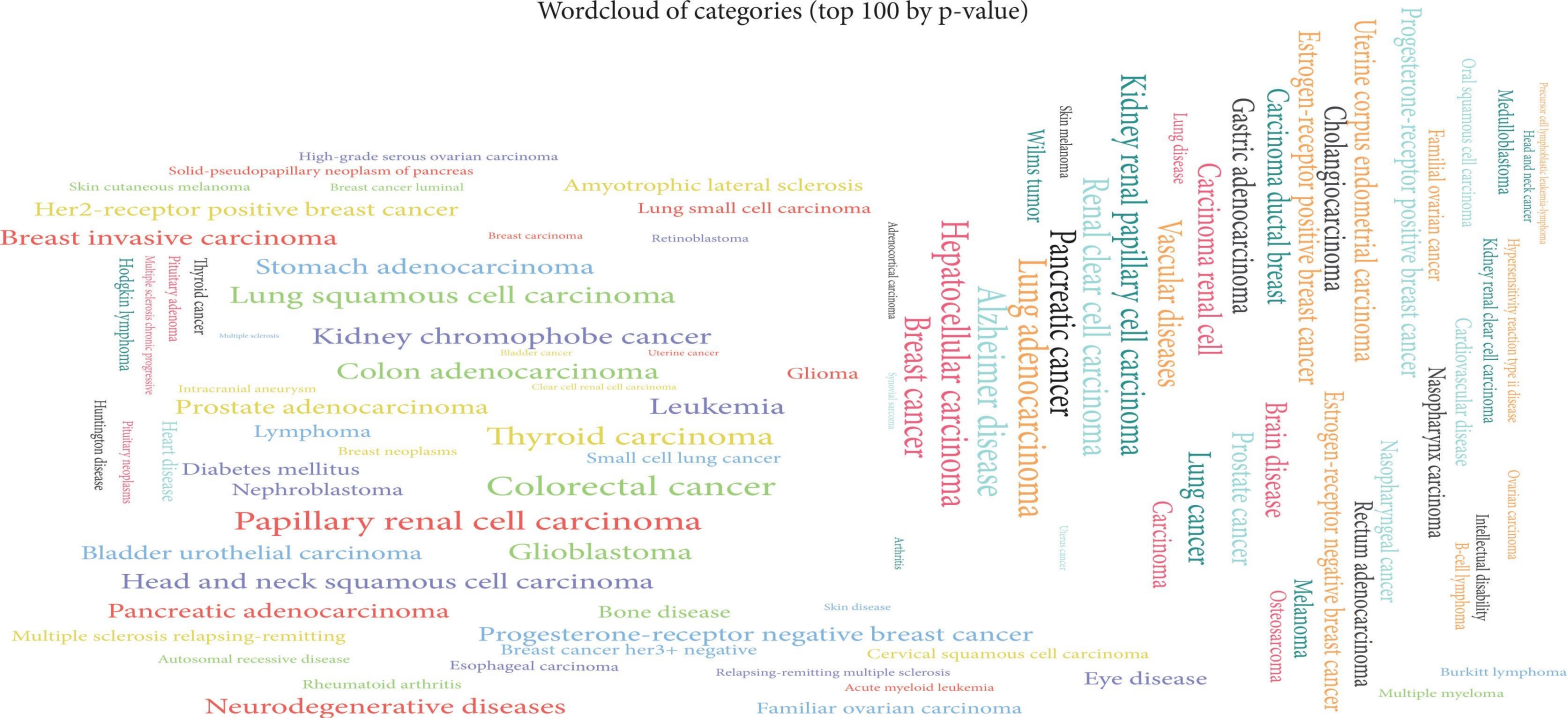
(b)

### 3.5. miRNA Enrichment Analysis and Identification of HCC‐Associated miRNAs

We used the miEAA database to analyze 97 differentially expressed miRNAs that regulate eight candidate genes from our miRNA data. These miRNAs are enriched in key pathways for HCC, such as cell proliferation and apoptosis regulation, and are visualized in a word cloud (Figure 2b).

### 3.6. Expression Pattern Analysis Through Box Plots

**Figure 3:**
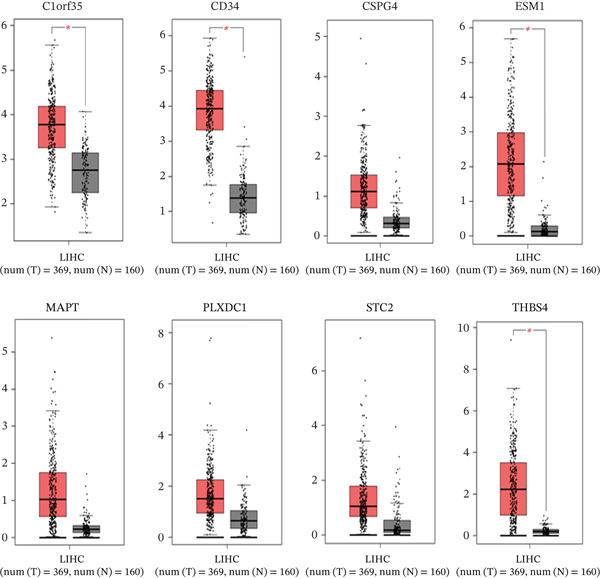
Expression analysis of eight ECM‐associated genes in liver cancer and normal tissues. Box plots from the GEPIA database comparing tumor and normal liver tissues. All eight genes were upregulated in HCC, with *CD34*, *C1orf35*, *ESM1*, and *THBS4* showing statistically significant differences (*p* < 0.05), while *MAPT*, *PLXDC1*, and *STC2* exhibited nonsignificant upward trends.

Moreover, we validated the expression profiles of the eight ECM‐associated genes using the GEPIA database and visualized their expression patterns in tumor and normal liver tissues through box plots. As shown in Figure 3, all eight genes exhibited higher expression levels in LIHC compared with normal tissues. Among them, *CD34*, *C1orf35*, *ESM1*, and *THBS4* showed statistically significant overexpression, whereas *MAPT*, *PLXDC1*, and *STC2* displayed nonsignificant upward trends. Despite the absence of statistical significance at this stage, these three genes were later confirmed to have strong prognostic relevance in survival analysis, supporting their potential as key biomarkers in HCC progression.

### 3.7. Survival Analysis of Identified Genes

The GEPIA database revealed that higher expression levels of *MAPT*, *PLXDC1*, and *STC2* were significantly associated with shorter overall survival (OS) (*p* < 0.05) in patients with liver cancer, indicating their potential prognostic importance. In contrast, *C1orf35*, *ESM1*, *CSPG4*, *CD34*, and *THBS4* did not show statistically significant associations with OS (Figure 4a). This analysis underscores that *MAPT*, *PLXDC1*, and *STC2* may serve as reliable prognostic indicators in HCC.

Figure 4Overall survival and ROC curve analysis. (a) Kaplan–Meier plots showing overall survival differences. Elevated expression of *MAPT*, *PLXDC1*, and *STC2* is significantly associated with shorter survival in HCC patients. (b) ROC curves illustrating diagnostic performance (*A* *U* *C* > 0.85) for all eight ECM‐associated genes. A higher AUC indicates stronger discriminatory ability between tumor and normal tissues (AUC, area under the curve; ROC, receiver operating characteristic).

**Figure:**
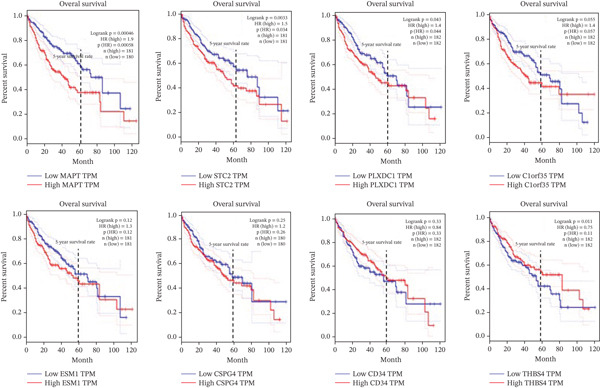
(a)

**Figure:**
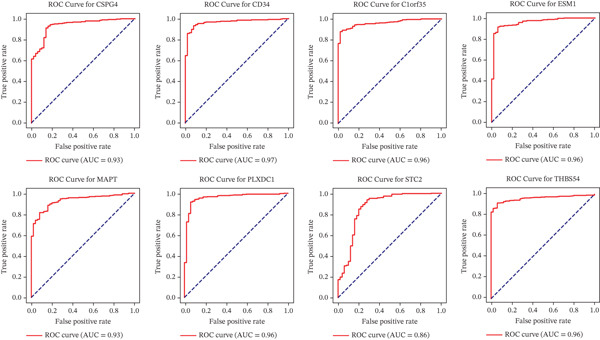
(b)

### 3.8. Include Individual ROC Curve Plots With Gene Names and AUC Values

The ROC curves illustrated the sensitivity and specificity of each gene as a classifier, and the AUC values provided a quantitative measure of their overall performance. Notably, genes with higher AUC values may warrant further investigations as potential biomarkers (Figure 4b and Table 3).

**Table 3:** ROC curve analysis.

Gene symbol	AUC
*CSPG4*	0.93
*CD34*	0.94
*C1orf35*	0.96
*ESM1*	0.96
*MAPT*	0.93
*PLXDC1*	0.96
*STC2*	0.86
*THBS4*	0.96

### 3.9. AFP Correlation With ECM‐Associated Genes

Among the eight ECM‐associated genes that were analyzed, statistically significant positive correlations with AFP levels were observed for *PLXDC1* (*ρ* = 0.20, *p* = 0.0039), *CSPG4* (*ρ* = 0.18, *p* = 0.0123), and *C1orf35* (*ρ* = 0.17, *p* = 0.0149). The remaining genes (*MAPT*, *STC2*, *THBS4*, *CD34*, and *ESM1*) exhibited no significant correlations (*p* > 0.05). These findings suggest that while certain ECM‐related markers may share partial clinical overlap with AFP, they likely capture distinct molecular aspects of HCC pathogenesis (Figure S1).

### 3.10. External Dataset Validation: Gene Consistency in Liver Cancer

To further strengthen the robustness and generalizability of the eight ECM‐associated biomarkers, we performed external validation using two independent paired tumor/adjacent RNA‐seq datasets: GSE104310 and GSE144269. GSE104310 contains paired HCC tumor and nontumor tissues profiled using Illumina HiSeq 2500, while GSE144269 comprises 70 paired samples from Mongolian HCC patients, representing an ethnically distinct high‐incidence population. Both datasets underwent VST normalization, and log2 fold‐changes were computed for direct comparison with TCGA‐LIHC. Consistent expression patterns were observed across all three cohorts. *CSPG4*, *CD34*, *ESM1*, *STC2*, and *THBS4* showed strong, reproducible upregulation in tumor tissues across datasets. *MAPT*, *PLXDC1*, and *C1orf35* demonstrated similar directionality and magnitude of change, confirming their stable upregulation across independent patient populations. Notably, the agreement among TCGA, GSE104310, and GSE144269 underscores that the observed transcriptional behavior of these ECM‐related genes is not cohort‐ or platform‐specific. The integration of two external GEO cohorts, one from Chinese patients and another from Mongolian patients, demonstrates cross‐population reproducibility, reinforcing the validity of the eight gene ECM signature. These findings collectively confirm that the identified biomarkers are consistently dysregulated across diverse HCC cohorts, sequencing technologies, and clinical sampling environments (Figure 5a).

Figure 5Validation and functional analysis of the candidate genes. (a) Line graph showing consistent expression patterns across the TCGA‐LIHC, GSE104310, and GSE144269 datasets. (b) Gene Ontology (GO) enrichment analysis of the eight candidate genes using the DAVID database. (c) Gene–gene interaction network generated by GeneMANIA, displaying functional associations among the candidate genes.

**Figure:**
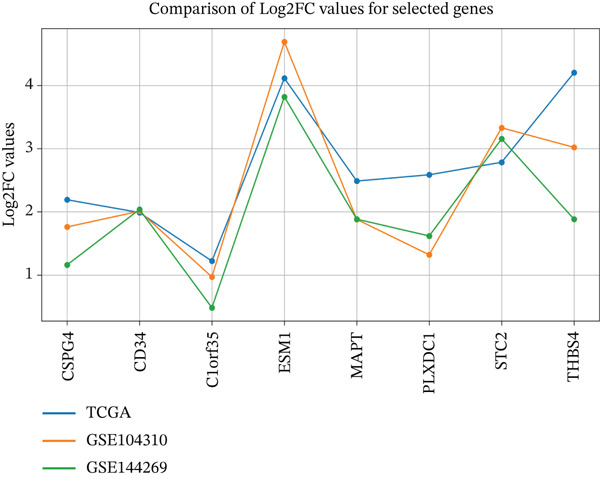
(a)

**Figure:**
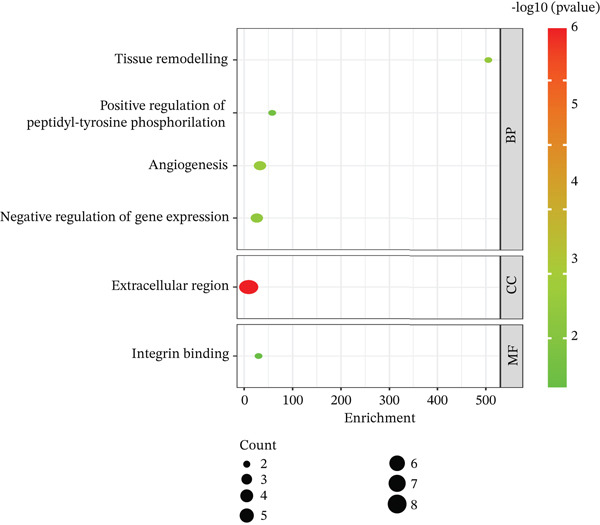
(b)

**Figure:**
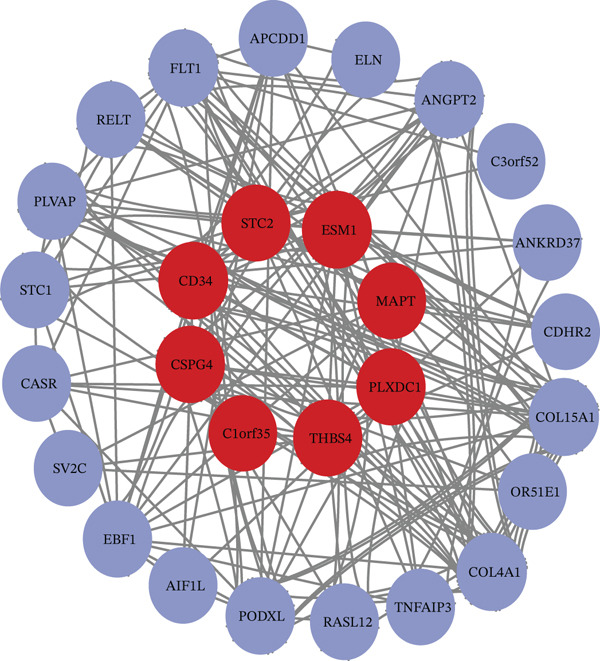
(c)

### 3.11. Functional Analysis of the Candidate Genes

To understand the potential function of candidate genes (*CSPG4*, *CD34*, *C1orf35*, *ESM1*, *MAPT*, *PLXDC1*, *STC2*, and *THBS4*), GO analyses were conducted using the DAVID databases (Figure 5b). Results suggested that these mRNAs were most significantly enriched in the GO terms of “tissue remodeling,” “positive regulation of peptidyl‐tyrosine phosphorylation,” “angiogenesis,” “negative regulation of gene expression,” “extracellular region,” and “integrin binding.” Additionally, GeneMANIA database analysis elucidates gene–gene functional interactions, providing insights into the collective behavior of these genes in the LIHC prognosis (Figure 5c).

### 3.12. Analyzing scRNA‐seq Data

**Figure 6:**
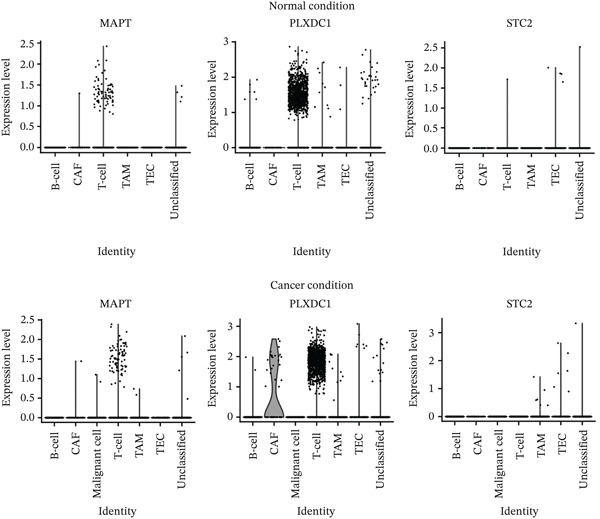
Violin plots of gene expression across cell subtypes. Violin plots illustrating single‐cell expression patterns of *MAPT*, *PLXDC1*, and *STC2* across cell subtypes in normal (upper panels) and cancerous (lower panels) liver tissues. *MAPT* shows increased expression in T cells of cancer samples, *PLXDC1* is enriched in cancer‐associated fibroblasts (CAFs), and *STC2* exhibits mild upregulation in tumor‐associated macrophages (TAMs) and tumor endothelial cells (TECs). Each dot represents a single cell; plots display expression distributions across clusters, revealing cell type–specific expression changes between normal and cancer conditions.

Integration of scRNA‐seq data from the selected normal and cancer liver samples generated two conditions, one representing normal tissue and the other capturing cancer tissue, each comprising distinct cell populations after quality filtering. Clustering analysis revealed multiple cell types within both conditions, underscoring the cellular heterogeneity of the liver microenvironment (Figure S2). Normal samples were primarily composed of T cells, accompanied by smaller clusters of cancer‐associated fibroblasts (CAFs), tumor‐associated macrophages (TAMs), tumor endothelial cells (TECs), and B cells. In contrast, cancer samples exhibited greater heterogeneity, characterized by prominent T cell and TAM clusters along with smaller populations of CAFs, TECs, B cells, malignant cells, and unclassified cells. These patterns reflect alterations in the tumor microenvironment and increased cellular diversity without a marked depletion of T cells. Analysis of gene expression across cell clusters revealed notable differences in three genes, *MAPT*, *PLXDC1*, and *STC2*, between normal and cancerous liver tissues. Violin plot visualization (Figure 6) showed that *PLXDC1* expression was elevated, particularly in CAFs, consistent with its association with stromal activation and ECM remodeling. *STC2* displayed mild upregulation within TAMs and TECs, suggesting a limited yet context‐specific role. *MAPT* demonstrated increased expression in T cell clusters of cancerous tissues compared to normal samples, indicating potential involvement in immune cell–associated signaling within the tumor microenvironment.

## 4. Discussion

Discovery of new biomarkers through noninvasive techniques is a crucial objective in early cancer diagnosis [25]. While numerous secretory proteins have been proposed as potential biomarkers, only a limited number have translated into clinical practice. The limited success arises from stringent selection criteria, including proteins that are selectively overexpressed in tumors, secreted into accessible fluids such as serum, and expressed minimally in normal tissues [26, 27]. In this regard, computational and machine learning algorithms, such as the RF model, have demonstrated significant potential for identifying biologically relevant features in high‐dimensional transcriptomic datasets [28, 29].

The present study employed a system‐level bioinformatics approach combined with machine learning to identify ECM‐associated biomarkers in HCC. Our analyses revealed eight ECM‐linked genes, *CSPG4*, *CD34*, *C1orf35*, *ESM1*, *MAPT*, *PLXDC1*, *STC2*, and *THBS4*, enriched in biological processes such as tissue remodeling, regulation of peptidyl‐tyrosine phosphorylation, angiogenesis, and negative regulation of gene expression. ROC curve analysis confirmed strong diagnostic power for all eight genes (AUC > 0.85), underscoring their potential for early detection. Among these, *MAPT*, *PLXDC1*, and *STC2* exhibited statistically significant associations with OS, identifying them as the core prognostic subset within this ECM‐associated signature. In contrast, *C1orf35*, *ESM1*, *CSPG4*, *CD34*, and *THBS4* were strongly upregulated but did not show significant OS associations, suggesting supportive roles in HCC progression rather than direct prognostic value.

*MAPT* (microtubule‐associated protein tau) has been implicated in cell proliferation, inflammation, and epithelial–mesenchymal transition (EMT)–related signaling [30]. Its expression varies with p53 mutation status and is elevated in aggressive tumors [31]. Geng et al. reported *MAPT* overexpression in HCC and its association with reduced OS [32]. Consistent with these findings, our study confirmed that high *MAPT* expression correlated with poorer survival. Functionally, *MAPT* may drive cytoskeletal reorganization and promote tumor cell motility via peptidyl‐serine phosphorylation, supporting its role as a key mediator of HCC progression.

*STC2* also demonstrated significant prognostic relevance. Previous studies have linked *STC2* overexpression to enhanced tumor growth, invasion, and chemoresistance in multiple cancers, including HCC [33, 34]. Upregulation of *STC2* promotes proliferation and colony formation, while its silencing induces cell cycle arrest at G0/G1 [35, 36]. Our data corroborate these findings, showing that elevated *STC2* expression associates with shorter OS in HCC patients. *PLXDC1*, also known as tumor endothelial marker 7 (TEM7), emerged as a major ECM‐linked prognostic gene. It acts as a mediator of angiogenesis and stromal remodeling [37]. Geng et al. and Meier et al. observed its upregulation in gastrointestinal and hepatic cancers [32, 38]. In our dataset, *PLXDC1* was significantly correlated with shorter OS, reinforcing its prognostic role in HCC. Mechanistically, *PLXDC1* expression has been linked to macrophage polarization toward the immunosuppressive M2 phenotype [39] and immune evasion in HCC [18], indicating its contribution to tumor microenvironment modulation and angiogenic activity.

Although *C1orf35*, *ESM1*, *CSPG4*, *CD34*, and *THBS4* did not exhibit significant survival associations, each contributes functionally to ECM remodeling and tumor progression. *C1orf35* has been identified as an oncogene promoting c‐MYC transcription and cell proliferation [38, 40]. *ESM1* plays an established role in angiogenesis and has been associated with the macrotrabecular‐massive HCC subtype [41–43]. *CSPG4* (NG2) participates in cell adhesion and metastasis through its interaction with collagen type VI [17, 44] and predicts poor outcomes in HCC ([45]). *CD34* marks endothelial activation during neovascularization in HCC [46, 47], while *THBS4*, a calcium‐binding glycoprotein, regulates tumor adhesion and migration via the ITGB1/FAK/PI3K/AKT axis [48, 49]. The upregulation of these five genes reinforces their collective diagnostic value and their role in ECM restructuring within the tumor milieu.

Validation using the GSE104310 and GSE144269 datasets confirmed consistent expression profiles for all eight genes, supporting reproducibility across platforms. scRNA‐seq further clarified the spatial expression of the three prognostic genes within the tumor microenvironment. *MAPT* was markedly increased in T cells, suggesting a potential influence on immune cell–mediated ECM remodeling and tumor–immune interactions. *PLXDC1* showed strong enrichment in CAFs, indicating its involvement in fibroblast activation, ECM deposition, and angiogenesis. *STC2* exhibited mild upregulation in TAMs and TECs, implying a role in angiogenic and inflammatory signaling. These cell type–specific patterns highlight *MAPT* and *PLXDC1* as central mediators of microenvironmental reprogramming in HCC, while *STC2* may exert a secondary modulatory effect. In addition, the correlation analysis between AFP levels and ECM‐associated genes revealed only weak associations for *PLXDC1*, *CSPG4*, and *C1orf35*, suggesting that these biomarkers may act independently of AFP and could provide complementary diagnostic value in HCC.

Pharmacological targeting of ECM‐associated genes holds therapeutic promise for HCC. Recent network pharmacology approaches have identified active compounds from traditional Chinese medicine that modulate ECM remodeling pathways, potentially synergizing with our biomarkers [50]. Similarly, advanced knowledge graphs integrating drug–target interactions could prioritize inhibitors against *PLXDC1/STC2/MAPT* [51]. High‐throughput chemical screening platforms further enable discovery of compounds modulating these ECM signatures [52], supporting drug repurposing strategies for our prognostic subset.

Collectively, this study focused on identifying and biologically characterizing ECM‐related genes. The RF algorithm provided a robust feature selection approach that ranked gene importance and ensured internal validation of the identified biomarkers. Each candidate gene was subsequently evaluated independently through ROC and Kaplan–Meier survival analyses, confirming their diagnostic and prognostic relevance. However, constructing a multigene prognostic model, such as those described in integrative frameworks, could further refine prediction accuracy and reveal synergistic interactions among ECM‐associated genes [53]. Future studies integrating our ECM candidates in composite models will be valuable to complement the individual marker approach established in the present analysis.

Although the present study relies exclusively on bioinformatics analyses of publicly available datasets and lacks direct experimental validation, it provides a strong computational foundation for identifying ECM‐related biomarkers in HCC. Future investigations will involve experimental validation through quantitative PCR, immunohistochemistry, and functional assays to confirm the expression and mechanistic roles of the identified ECM‐associated genes, particularly *MAPT*, *PLXDC1*, and *STC2*, which exhibited significant prognostic associations. Such studies will be essential to translate these computational findings into clinically actionable biomarkers.

**Nomenclature**
LIHC: liver hepatocellular carcinoma
HCC: hepatocellular carcinoma
ECM: extracellular matrix
TCGA: The Cancer Genome Atlas
OS: overall survival
AUC: area under the curve
ROC: receiver operating characteristic
DEGs: differentially expressed genes

## Author Contributions

P.A.S.: conception of the research, collected the data, data analysis, and interpretation; E.R.: drafting and revising the manuscript; A.A.J.: collected the data, data analysis, and interpretation; A.F. and Z.H.: drafting the manuscript; M.H.: revising the manuscript; M.V.: supervision.

## Funding

No funding was received for this manuscript.

## Disclosure

M.V. provided final approval of the manuscript. All authors reviewed the manuscript.

## Conflicts of Interest

The authors declare no conflicts of interest.

## Supporting information


**Supporting Information** Additional supporting information can be found online in the Supporting Information section. Table S1: Detailed specific parameters of quality control (QC). Figure S1: Correlation of ECM‐associated gene expression with AFP levels in TCGA‐LIHC. Scatter plots showing the relationship between AFP concentration (log2‐transformed, *g*/*L* + 1) and mRNA expression (RNA‐Seq TPM) for eight ECM‐associated genes (*CSPG4*, *CD34*, *C1orf35*, *ESM1*, *MAPT*, *PLXDC1*, *STC2*, and *THBS4*). Each panel displays Spearman’s and Pearson’s correlation coefficients with corresponding *p* values. Significant positive correlations were detected for *PLXDC1*, *CSPG4*, and *C1orf35*, while other genes showed no significant association with AFP levels. Figure S2: UMAP and *t*‐SNE plots of single‐cell RNA‐seq data from normal and cancer liver tissues. Normal samples are dominated by T cells with smaller B cell, CAF, TAM, and TEC clusters. Cancer samples display greater heterogeneity, featuring prominent T cell and TAM clusters with smaller B cell, CAF, TEC, malignant, and unclassified clusters. These patterns reflect microenvironmental remodeling in cancer without significant T cell depletion.

## Data Availability

The data that support the findings of this study are available from the corresponding author upon reasonable request.
