# Supplementary material for: Extracellular Matrix–Associated Biomarkers for Hepatocellular Carcinoma: Insights From Machine Learning and Single‐Cell Analysis
Source: Int J Genomics. 2026 Feb 24;2026:6654142. doi: 10.1155/ijog/6654142 (PMC12932914; doi:10.1155/ijog/6654142)
Supplement: Supplementary file 1 — Supporting Information Additional supporting information can be found online in the Supporting Information section. Table S1: Detailed specific parameters of quality control (QC). Figure S1: Correlation of ECM‐associated gene expression with AFP levels in TCGA‐LIHC. Scatter plots showing the relationship between AFP concentration (log2‐transformed, g/L + 1) and mRNA expression (RNA‐Seq TPM) for eight ECM‐associated genes (CSPG4, CD34, C1orf35, ESM1, MAPT, PLXDC1, STC2, and THBS4). Each panel displays Spearman’s and Pearson’s correlation coefficients with corresponding p values. Significant positive correlations were detected for PLXDC1, CSPG4, and C1orf35, while other genes showed no significant association with AFP levels. Figure S2: UMAP and t‐SNE plots of single‐cell RNA‐seq data from normal and cancer liver tissues. Normal samples are dominated by T cells with smaller B cell, CAF, TAM, and TEC clusters. Cancer samples display greater heterogeneity, featuring prominent T cell and TAM clusters with smaller B cell, CAF, TEC, malignant, and unclassified clusters. These patterns reflect microenvironmental remodeling in cancer without significant T cell depletion. [file IJOG-2026-6654142-s001.docx]

Table.S1

Table legend: Detailed specific parameters of quality control (QC)

| **Dataset** | **Type** | **Sample ID** | **Min Cell** | **Min nFeature** | **Max nFeature** | **MT percentage** | **No. cell before QC** | **No. cell after QC** |
| --- | --- | --- | --- | --- | --- | --- | --- | --- |
| **GSE189903** | Cancerous | GSM5709307 | 3 | 81 | 1389 | 20 | 7305 | 7040 |
| **GSE189903** | Cancerous | GSM5709309 | 3 | 299 | 1289 | 20 | 7526 | 7128 |
| **GSE189903** | Cancerous | GSM5709314 | 3 | 92 | 2002 | 20 | 8991 | 8636 |
| **GSE189903** | Cancerous | GSM5709326 | 3 | 0 | 1664 | 20 | 6401 | 5960 |
| **GSE189903** | Cancerous | GSM5709332 | 3 | 0 | 4172 | 20 | 2109 | 1922 |
| **GSE189903** | Cancerous | GSM5709336 | 3 | 247 | 1238 |  | 7141 | 6677 |
| **GSE189903** | Adjacent Normal Tissue | GSM5709305 | 3 | 0 | 2140 | 20 | 786 | 711 |
| **GSE189903** | Adjacent Normal Tissue | GSM5709316 | 3 | 231 | 1919 | 20 | 11344 | 10822 |
| **GSE189903** | Adjacent Normal Tissue | GSM5709324 | 3 | 453 | 1365 | 20 | 8179 | 7781 |
| **GSE189903** | Adjacent Normal Tissue | GSM5709329 | 3 | 130 | 1920 | 20 | 10574 | 10062 |

**
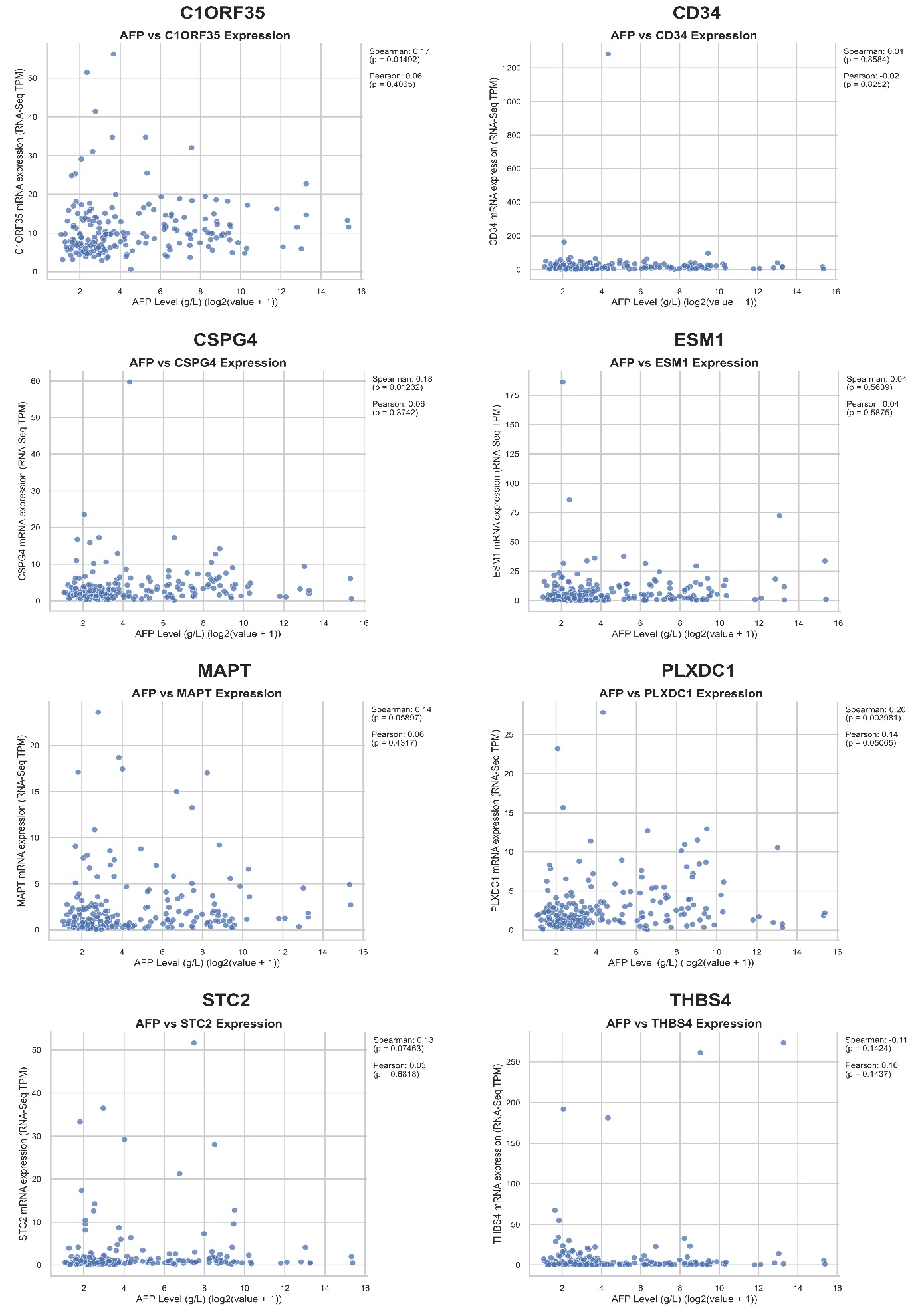
**

**Fig.S1.** Correlation of ECM-associated gene expression with AFP levels in TCGA-LIHC.
Scatter plots showing the relationship between AFP concentration (log2-transformed, g/L + 1) and mRNA expression (RNA-Seq TPM) for eight ECM-associated genes (*CSPG4*, *CD34*, *C1orf35*, *ESM1*, *MAPT*, *PLXDC1*, *STC2*, and *THBS4*). Each panel displays Spearman’s and Pearson’s correlation coefficients with corresponding p-values. Significant positive correlations were detected for *PLXDC1*, *CSPG4*, and *C1orf35*, while other genes showed no significant association with AFP levels.


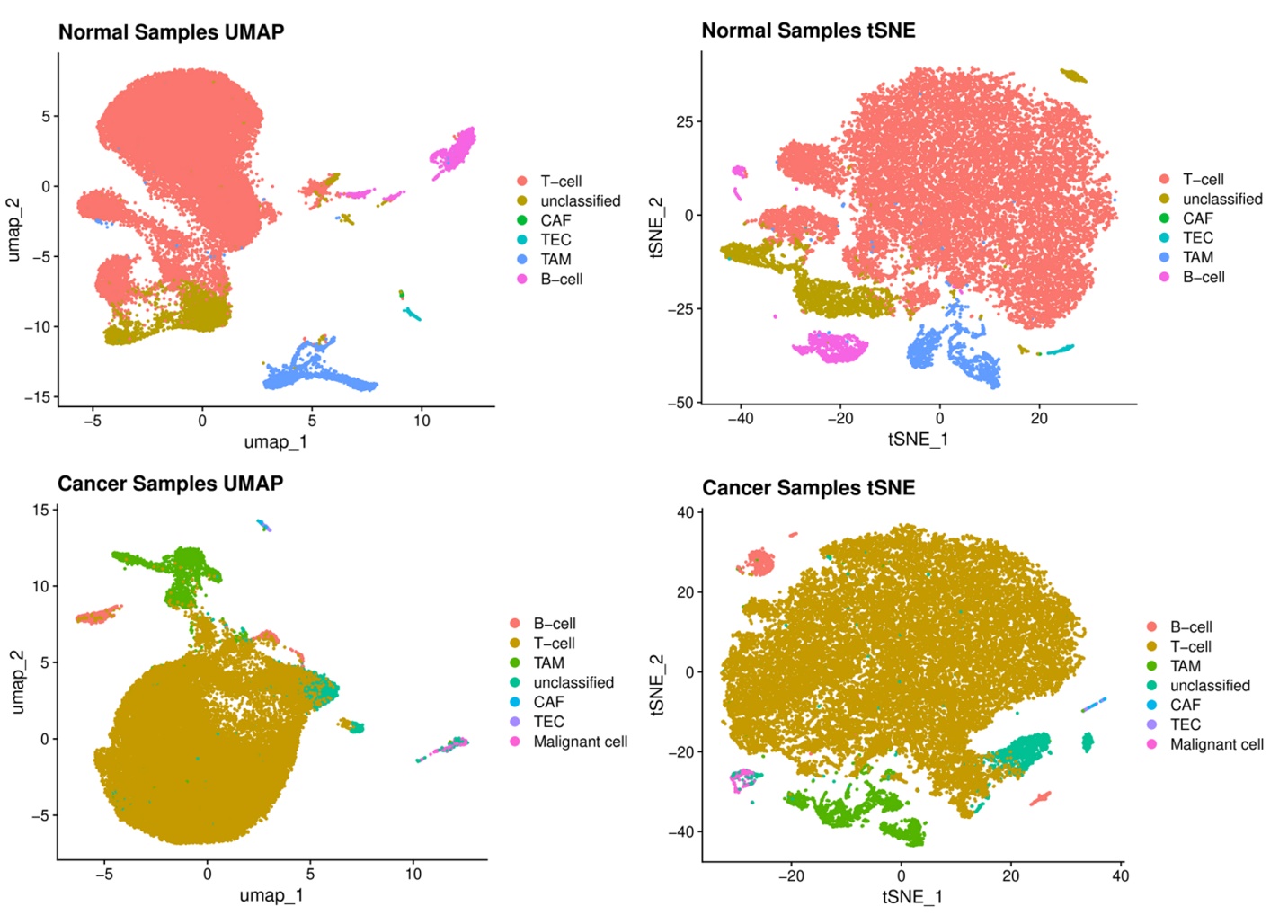


**Fig.S2** UMAP and t-SNE plots of single-cell RNA-seq data from normal and cancer liver tissues. Normal samples are dominated by T cells with smaller B-cell, CAF, TAM, and TEC clusters. Cancer samples display greater heterogeneity, featuring prominent T-cell and TAM clusters with smaller B-cell, CAF, TEC, malignant, and unclassified clusters. These patterns reflect microenvironmental remodeling in cancer without significant T-cell depletion.
